# Cardiovascular Autonomic Profile in Women With Premenstrual Syndrome

**DOI:** 10.3389/fphys.2018.01384

**Published:** 2018-10-01

**Authors:** Rimma Koifman, Lior Dayan, Jacob N. Ablin, Giris Jacob

**Affiliations:** Department of Internal Medicine F, J. Recanati Autonomic Dysfunction Center, Tel Aviv Sourasky Medical Center, Faculty of Medicine, Tel Aviv University, Tel Aviv, Israel

**Keywords:** autonomic nervous system, premenstrual syndrome, baroreflex sensitivity, head-up tilt test, spectral analysis

## Abstract

**Introduction:** The premenstrual syndrome (PMS) is a constellation of somatic and psychogenic symptoms that appear during late luteal (LL) phase of the menstrual cycle. Since many symptoms could be related to the autonomic nervous system, we hypothesized that the sympathetic nervous system is perturbed in PMS.

**Methods:** The cardiovascular autonomic profile of nine women with PMS (30.4 ± 2.5 years) were compared to that of nine healthy controls (30 ± 2.5 years) during their early follicular (EF) and LL phases of the menstrual cycle. Plasma norepinephrine (NE) concentrations, power spectral analysis of heart rate and systolic blood pressure (BP), and baroreflex sensitivity (BRS) were assessed during recumbency and a head-up tilt (HUT). Cardiovascular responsiveness to α1- and β-adrenoreceptor agonists (phenylephrine and isoproterenol, respectively) were also assessed.

**Results:** In the LL phase, the plasma NE concentrations in women with PMS during recumbency and a HUT were lower than those in women without PMS [180 ± 30 vs. 320 ± 50 pg/ml; *p* = 0.04 (recumbent), and 480 ± 70 vs. 940 ± 180 pg/ml: *p* = 0.02 (HUT)]. In the LL phase, the dose of phenylephrine required to increase systolic BP by 15 mmHg in women with PMS was significantly greater than that in women without PMS (202 ± 30 μg vs. 138 ± 20 μg; *p* = 0.02). Sympathetic and vagal cardiac control indices were comparable in the two groups in the menstrual phases. In women with PMS, the value of LF_*SBP*_ in the LL phase was lower than that in the EF phase (0.98 ± 0.2 vs. 1.77 ± 0.4 mmHg^2^, *p* = 0.04). The increase in LF_*SBP*_ in women with PMS in the LL phase during HUT was greater than that in the controls, 5.2 ± 0.9 vs. 3.1 ± 0.5 mmHg^2^, *p* = 0.045, and this increase was associated with a significant decrease in BRS.

**Conclusion:** In women with PMS without psychogenic symptoms, the sympathetic control of their circulation is not dominant during the LL phase of their menstrual cycle.

## Introduction

The premenstrual syndrome (PMS) is the most common functional disorder in women during their reproductive years (Ismaili et al., 2016). Women with PMS can have different profiles of cyclic symptoms, which characteristically appear in the late luteal (LL) phase of the menstrual cycle and subside within a few days after the onset of menstruation (Halbreich et al., 1993; Redei and Freeman, 1995).

Notwithstanding the protean nature of the symptoms, they can broadly be categorized into two groups. The first group comprises behavioral and psychological symptoms, such as anxiety, depression, mood swings, cloudy thought, and irritability. The second group includes a variety of somatic symptoms, which can be arbitrarily separated into two subgroups, volume-related and sympathetic-related (Rosenfeld et al., 2008; Ismaili et al., 2016).

Since the severity of the cognitive–affective symptoms of premenstrual disorders has tended to overshadow the investigational approach to PMS, psychiatrists have recently recommended that premenstrual disorder can be listed as a distinct category in DSM-5 (premenstrual dysphoric disorder—PMDD) (Epperson et al., 2012). Accordingly, investigations into PMDD have focused on the syndrome’s psycho-behavioral profile, while partially neglecting the pathophysiology of the somatic symptoms. On the other hand, cases in which significant somatic symptoms present together with mild to moderate behavioral and affective symptoms are usually diagnosed as PMS and treated by gynecologists (Biggs and Demuth, 2011).

We have previously reported on the pathophysiology of the somatic volume-dependent symptoms of PMS. We found that the edema-related symptoms during the late LL of the menstrual cycle are partially related to increased plasma levels of the fluid regulatory hormones, such as aldosterone, and elevated plasma renin activity (Rosenfeld et al., 2008). However, the hemodynamic physiology and pathophysiology of the sympathetic-related symptoms, such as dizziness, pre-syncope, palpitation, nausea, and others remain unexplored in women with PMS.

Our knowledge on the activity of the autonomic nervous system in women with PMS has been usually obtained from women with PMDD (Landen et al., 2004; Matsumoto et al., 2007; Klatzkin et al., 2010). Notably, the diagnosis of PMDD is based on the presence of symptoms, such as panic and depression, which are well-known to be increased in the presence of sympathetic overactivity, irrespective to the presence or absence of premenstrual disorders (Landen et al., 2004; de Zambotti et al., 2013).

Against this background, we hypothesized that the sympathetic control of the cardiovascular system is perturbed in women with PMS (without any psychiatric syndromes) and this could account for their deranged hemodynamics, and the somatic symptoms could be attributed to overactivity of the sympathetic nervous system.

In order to test this hypothesis, we stringently selected women with only somatic signs of PMS and without any significant psychiatric comorbidity for the investigation.

## Materials and Methods

### Subjects

The study’s participants were recruited through community advertisements. Although more than 100 women responded to the advertisement, only nine women with PMS fulfilled our stringent inclusion criteria (see later). The control group comprised nine age-matched women without PMS. The diagnosis of PMS was determined according to the Shortened Premenstrual Assessment Form (SPAF) (Allen et al., 1991), a questionnaire that classifies premenstrual symptoms (physical and mood) into ten categories on a scale of 1–6.

Women were diagnosed as having PMS if they scored at least five of the premenstrual symptoms as severe (≥5) in the subscales of the SPAF questionnaire. Women in the control group were considered as not having PMS if they scored less than 2 for each of the 10 premenstrual symptoms in the subscales of the SPAF questionnaire. Participants completed the SAPF at least three menstrual cycles before the study. All participants were apparently completely healthy, and none had any history of alcohol or drug abuse, smoking, or used oral contraception within the 3 months prior to the start of the study. All participants were aged between 20 and 45 years, had a normal body mass index (BMI) (19–25 kg/m^2^), and had normal thyroid function.

### Experimental Design

The study protocol was approved by the Tel Aviv “Sourasky” Medical Center’s Ethics Review Board (IRB-1628), and the investigation was done according to the principles of the Declaration of Helsinki. On recruitment, each subject signed a consent form to participate in the investigation following an explanation of its purpose, nature, and potential risks. After signing the informed consent form, the medical history of each participant was recorded before undergoing a physical examination and completing an extensive questionnaire, which was designed to assess autonomic symptoms. Each participant was asked to refrain from (a) eating any food or beverages, which contained caffeine, another methylxanthine, or any other central nervous system stimulant, (b) smoking, and (c) doing any exercise at least 24 h before the study.

All investigational procedures were performed after overnight fasting in a quiet, partially darkened, and air-conditioned room whose ambient temperature was ∼24°C at the Recanati Autonomic Dysfunction Center.

Each woman was studied at two different time points within one menstrual cycle (Hirshoren et al., 2002):

• The early follicular (EF) phase—day 3–4 (low plasma estrogen and low plasma progesterone levels)
• The late LL phase—days 26–27 (withdrawal phase of both hormones)

These time points were selected because most premenstrual symptoms appear during the late LL phase, and subside a few days after the EF phase (Campagne and Campagne, 2007).

#### Instrumentation

On the day of testing, each woman was asked to void before being placed in a recumbent position on a tilt table and insertion of an 18G intravenous catheter for blood sampling and drug administration. Heart rate (HR) and beat-to-beat radial arterial blood pressure (BP) were continuously monitored and non-invasively used a three-lead electrocardiograph and a tonometric BP monitor (Colin CBM-7000, Colin Corp., Komaki City, Japan) whose outputs were displayed on a computer screen and on a thermal array recorder (TA-6000, Gould, Valley View, OH, United States). Data were digitized at 500 Hz by an analog-to-digital converter using the Windaq data acquisition software (WinDaq, version 2.27, DataQ Instruments, Akron, OH, United States). Data were stored on the hard disk of a personal computer for off-line analysis using locally developed software. BP was also measured using an oscillometric digital BP monitor with an upper arm cuff to calibrate the tonometric BP monitor before each procedure. Forearm blood flow was assessed using a strain gauge plethysmograph (ECR5, D. E. Hokanson, Inc., Bellevue, WA, United States).

#### Blood Sampling and Tilt Protocol

After instrumentation, each subject was allowed to rest in the recumbent position for 30 min. At the end of this period, data for power spectral analysis of the R-R interval (RRI) and BP were collected for 6 min (see below). Blood was then drawn for determining the plasma norepinephrine (NE) concentration and the plasma levels of the ovarian steroid hormones, estradiol (E2) and progesterone (P). Each subject was then tilted head-up at 60° for 20 min, and data for power spectral analysis of RRI and BP were collected for 6 min. Then, a blood sample was drawn for determining the plasma NE concentration at the end of the head-up tilt (HUT).

The plasma NE concentrations were measured using a previously described method (Jacob et al., 1999). A 5-ml blood sample was collected in a plastic syringe and then immediately transferred to a chilled EDTA vacuum tube, which was immediately placed in crushed ice. Blood was separated by refrigerated centrifugation at −4°C, stored at −70°C, and assayed within 6 months.

#### Pharmacologic Testing

Baseline measurements of HR and BP were made in each subject after a 20-min rest in a recumbent position. Four or five bolus doses of the drugs, isoproterenol (ISOP), and phenylephrine (PHE), were administered intravenously at 5–10-min intervals between each bolus in order to achieve the end points and to allow for each study parameter to return to its baseline value. Specifically, the initial dose of ISOP was 0.125 mg and incrementally increased until the HR increased by 25 beats/min (bpm) or the systolic BP decreased by 15 mmHg. The initial dose of PHE was 12.5 μg and incrementally increased until the systolic BP increased by 25 mmHg. Dose-response curves for ISOP and PHE were constructed in order to determine (a) the ISOP_15_, the ISOP dose that increased the HR by 15 bpm and (b) the PHE_15_, the PHE dose that increased systolic BP by 15 mm Hg. The ISOP_15_ was used as an index of β-adrenoreceptor sensitivity, and PHE_15_ was used an index of α_1_-adreneroceptor sensitivity (Jacob et al., 1999).

#### Power Spectral Analyses

Power spectral analyses of the RRI and the beat-to-beat systolic BP variability were done using the Welch periodogram method for power spectral density calculation. A band pass filter was used to filter out the respiratory signal and noise reduction. A Hanning window in the time domain was adopted to attenuate spectral leakage (512 samples). Two subsets of the frequency domain were used for RR and systolic BP variability, a low-frequency (LF_*RR*_: 0.04–0.14 Hz) band and a high-frequency (HF_*RR*_: 0.15–0.4 Hz) band. The LF and HF were also measured in normalized units, which represent the relative value of each power component in proportion to the total power minus the very LF component. Baroreflex sensitivity (BRS) and sequential BRS were calculated from the time domain data of the RRI and the beat-to-beat systolic BP variability (6-min recording) using a previously described protocol (Pagani et al., 1986; Furlan et al., 2000, 2001). The BRS, both the BRS_−seq_ and the BRS_+seq_ slopes (in milliseconds (ms)/mmHg) represent the corresponding increase and decrease in systolic BP and RR, respectively. For this analysis, the computer software selected all sequences of three or more successive heart beats in which there were concordant increases (BRS_+seq_) or decreases (BRS_−seq_) in the systolic BP and RRI. The minimal change for systolic BP had to be 0.5 mmHg and 4 ms for the RRI. A linear regression (*r* > 0.85) was applied to each of the sequences, and an average regression slope was calculated for the sequences that were detected during each recording period (Bertinieri et al., 1985).

### Statistical Analysis

A power analysis was used to calculate sample size. For this analysis, the statistical power was set at 80%, as used for mechanistic studies, the value of alpha was set at 0.05 for two-tailed paired testing, and the value of beta was set at 0.20. The calculation was based on the mean ± SD of our main study parameters, such as plasma NE concentrations and the value of PHE_15_.

Data were analyzed using Excel (Microsoft, Redmond, WA, United States) and Prism version 6.02 for Windows (GraphPad Software Inc., La Jolla, CA, United States). All results are expressed as mean ± SEM and statistical significance was set at 5%. Each parameter was tested for normality by using the Kolmogorov–Smirnov goodness-of-fit test. Parametric data were analyzed using a paired and unpaired two-tailed *t*-tests, for intra- and intergroup comparisons. Non-parametric data were analyzed using the Wilcoxon matched pairs test for paired data and the Mann–Whitney *U* test for unpaired data. A mixed model of repeated measures two-way analysis of variance (ANOVA) was used to assess the significance of the interaction of the menstrual phase per group and the effect of menstrual phase on measured parameters. For *post hoc* analysis, we used the Bonferroni’s test for parametric data. Contingent data were compared by using the Chi squared two-tailed test. Linear regression analysis was used to assess the dose–response relationship of ISOP_15_ and PHE_15_ and for the eventual correlation between different measured parameters.

## Results

The mean and range of ages and BMIs of the women with PMS and the women without PMS (control) were not significantly different from each other [(30.4 ± 2.5 (range: 21–40) and 30 ± 2.5 (range: 21–43) years, and 21 ± 0.7 and 24 ± 1.5 kg/m^2^, *p* = 0.055), respectively]. The duration of the menstrual cycle in the two groups of women was regular and the cycle lengths, calculated over three consecutive cycles, were comparable in both groups (29.5 ± 0.4 days in the women with PMS and 28.5 ± 0.5 days in the women without PMS (controls). The plasma P and E2 levels, the systolic and diastolic BPs, and the HRs during the LF and LL phases were comparable in the two groups of women (**Table 1**). **Table 2** summarizes the autonomic symptom profile of the two groups of women. As expected, the autonomic symptoms are more prevalent in the women with PMS than in the women without PMS.

**Table 1:** Hemodynamic and hormonal profile of women without premenstrual syndrome (CON) and women with PMS during the early follicular phase (EF) and late luteal (LL) phase of the menstrual cycle.

	CON		PMS	
	EF	LL	EF	LL
Plasma estrogen levels, pmol/l	287 ± 64	364 ± 104	124 ± 12	446 ± 55^∗^
Plasma progesterone levels, nmol/l	1.35 ± 0.25	17.65 ± 3.5^∗^	1.60 ± 0.25	29.5 ± 6^∗^
Estrogen/progesterone ratio	110 ± 32	21 ± 4	414 ± 150	23.5 ± 4
Systolic BP, mmHg	103 ± 2	107 ± 4	106 ± 2	109 ± 2
Diastolic BP, mmHg	62 ± 1	61 ± 2	62 ± 2	62 ± 2
Heart rate, bpm	62 ± 3	66 ± 2	60 ± 2	66 ± 2
Forearm blood flow, ml/min/dl	2.2 ± 0.20	3.3 ± 0.25^∗^	3.2 ± 0.8	2.7 ± 0.2^#^
Forearm vascular resistance mmHg/ml^−1∗^min^−1∗^dl^−1^	34 ± 4	23.5 ± 1.8^∗^	34 ± 6	29 ± 2^#^

*Data are displayed as mean ± SEM.^∗^p < 0.05, paired data between the menstrual phases EF and LL into each group, ^#^p < 0.05, unpaired data between the two groups in each menstrual phase. A two-tailed t-test was used for comparing the parametric data. Wilcoxon matched pairs test for paired data and Mann–Whitney U test for unpaired data were used for comparing the non-parametric data.*

**Table 2:** Commonly reported symptoms of women with premenstrual syndrome (PMS) and women without PMS (CON).

	PMS (*n* = 9)	CON (*n* = 9)	*P* value
Cloudy thought	6	1	0.015
Forgetfulness	6	0	0.003
Sleep disorder	1	0	ns
Fatigue	7	3	0.058
Emotional liability	6	2	0.056
Irritability	9	1	<0.001
Anxiety	9	1	<0.001
Syncope	0	0	ns
Presyncope	3	0	0.058
Dizziness	2	1	ns
Palpitation	3	0	0.058
Chest discomfort	1	1	ns
Nausea	4	2	ns
Headache (tension)	5	1	0.05
Hyperhidrosis	3	2	ns
Tremor	0	0	ns
Hyperventilation syndrome	0	0	ns
Flushing	1	0	ns
Diarrhea (during EF)	6	4	ns
Constipation	3	0	0.058
Heat intolerance	5	2	ns
Alcohol intolerance	0	1	ns

*Differences between groups were tested by using the Chi-squared test. ns, not significant.*

### Plasma NE Concentrations

**FIGURE 1:**
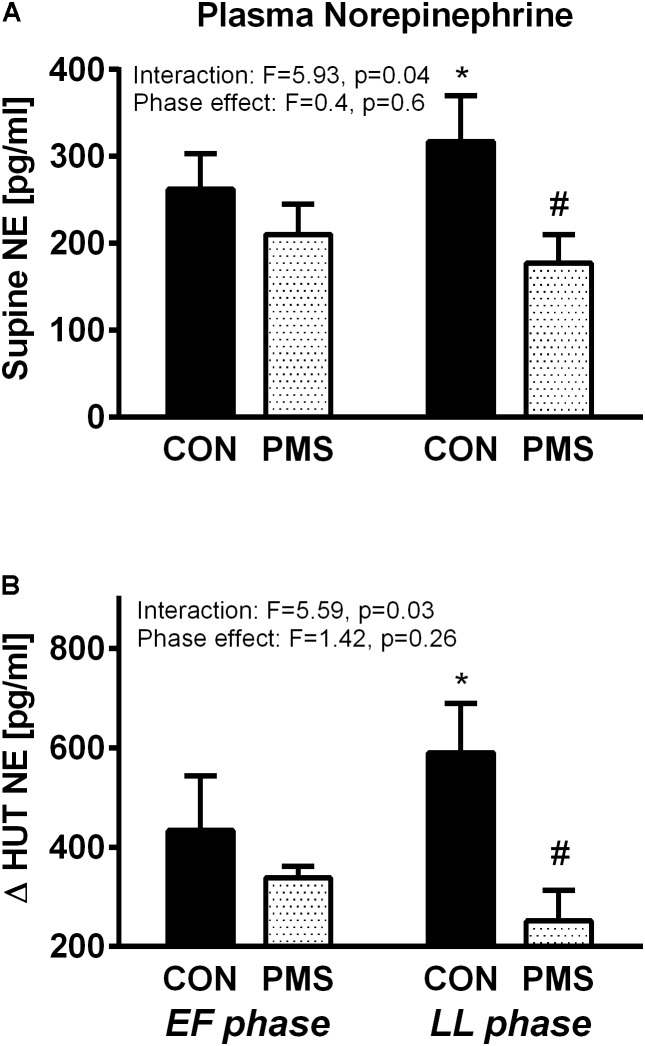
Plasma norepinephrine concentration in women without premenstrual syndrome (PMS; CON) and with PMS in a recumbent position (**A**, Upper panel) and the change in plasma norepinephrine levels during a 60° head-up tilt (**B**, Lower panel) in the early follicular and late luteal phases of the menstrual cycle. A mixed model of two-way ANOVA for repeated measures was used to assess the significance of menstrual phase–group interaction and menstrual phase effect. Bonferroni’s test was used for *post hoc* analysis. ^∗^*p* < 0.05, paired data between the menstrual phases EF and LL into each group, ^#^*p* < 0.05, unpaired data between the two groups in each menstrual phase.

**Figures 1A,B** display the plasma NE concentrations of the two groups of women in the EF and LL phases of their menstrual cycle when in a recumbent position and the increase during a 60° HUT. The control women, but not the women with PMS, have higher plasma norepinephrine concentrations during the LL phase than in the EF phase when recumbent (**Figure 1A**) and during the HUT (**Figure 1B**). The women with PMS had significantly lower plasma NE concentrations than the control women during the LL phase when recumbent (180 ± 30 vs. 320 ± 50 pg/ml, *p* < 0.05) and during a 60° HUT (480 ± 70 vs. 940 ± 180 pg/ml, *p* < 0.05). The interactions between menstrual phase–group, as tested by two-way ANOVA for repeated measures were statistically significant. For plasma NE concentration when recumbent, *F* = 5.93, *p* = 0.04, and for the change in plasma NE concentrations during a 60° HUT, *F* = 5.96, *p* = 0.03. There was no effect of the menstrual phase on data, *F* < 1.5 and *P* > 0.5 for both postures.

### Adrenoreceptor Sensitivity

**FIGURE 2:**
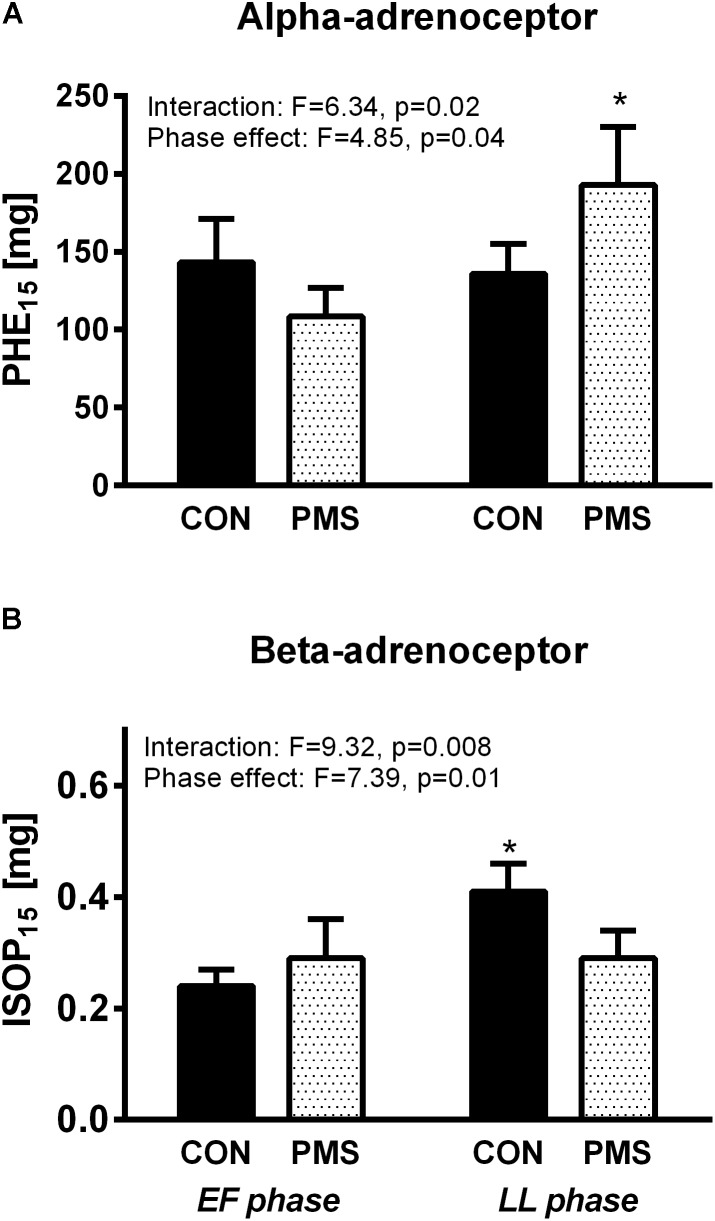
Responses of women without premenstrual syndrome (CON) and with PMS to graded intravenous boluses of phenylephrine (selective α1-adrenoceptor agonist), as represented by the PHE_15_ value (**A**, Upper panel): the phenylephrine dose which increased the systolic blood pressure by 15 mmHg (**A**, Upper panel), and isoproterenol (β-adrenoceptor agonist), as represented by the ISOP_15_ value (**B**, Lower panel): the isoproterenol dose which increased the heart rate by 15 beats/minute in the early follicular and late luteal phases of the menstrual cycle. A mixed model of two-way ANOVA for repeated measures was used to assess the significance of menstrual phase–group interaction and menstrual phase effect. Bonferroni’s test was used for *post hoc* analysis. ^∗^*p* < 0.05, paired data between the menstrual phases EF and LL into each group.

The values of PHE_15_ and ISOP_15_ in the EF and LL phases in the menstrual cycle of the two groups of women in a recumbent position and during a 60° HUT are displayed in **Figures 2A,B**. The interactions between the menstrual phase–group and the menstrual phase effect, as tested by two-way ANOVA for repeated measures, were statistically significant for the values of PHE_15_ and ISOP_15_ (**Figures 2A,B**). During the EF phase, the PHE_15_ values were not significantly different in the two groups of women. Additionally, the PHE_15_ values were not significantly different in the control group during the EF and LF phases. However, the women with PMS were less sensitive to PHE and had a significantly higher PHE_15_ value during the LL phase than that in the EF phase (138 ± 20 μg vs. 202 ± 30 μg; *p* < 0.05, **Figure 2A**). The responses of the women with PMS and the women without PMS to ISOP during the EF phase were not significantly different, as expressed by ISOP_15_ values (0.3 ± 0.03 μg vs. 0.24 ± 0.03 μg, respectively). The ISOP_15_ value was unchanged in women with PMS during LL phase, whereas the women without PMS became less sensitive to ISOP, as expressed by the higher ISOP_15_ value (**Figure 2B**).

### Power Spectral Analysis and Baroreflex Sensitivity

**Table 3** summarizes the results of the power spectral analyses. Irrespective of the menstrual phase, all HR variability parameters, namely the LF_*RR*_, the HF_*RR*_, and the LF_*RR*_:HF_*RR*_ ratio were comparable when subjects were recumbent. In the women with PMS, the LF_*SBP*_, the index of vascular sympathetic tone, was significantly lower during LL phase than that during the EF phase when they were recumbent [results of the two-way ANOVA revealed a significant phase–group interaction, *F* = 6.21, *p* = 0.025, but not a significant menstrual phase effect (*F* < 1.0, *p* = 0.30)]. Expectedly, the variance, LF_*RR*_ and HF_*RR*_ values during a 60° HUT were significantly lower than those when these women were recumbent. However, the decrease in the HF_*RR*_ was significantly less pronounced during EF phase than in the LL phase (271 ± 82 ms^2^ during the LL phase and 130 ± 30 ms^2^ during the EF phase, *p* < 0.05). Although the LF_*RR*_:HF_*RR*_ ratio and the LF_*SBP*_ significantly increased in the two groups of women in the two menstrual phases during the HUT, the increase in the LF_*SBP*_ was greater during LL phase than in the EF phase in the women with PMS, 5.2 ± 0.9 vs. 3.1 ± 0.5 mmHg^2^, *p* < 0.05 [results of the two-way ANOVA revealed a non-significant phase–group interaction (*F* = 1.2, *p* = 0.25), but the menstrual phase effect was statistically significant (*F* = 5.75, *p* = 0.03)]. **Table 3** displays the baroreflex sensitivity indices when the women were recumbent and during a 60° HUT. When recumbent, we did not detect any significant differences in the sensitivity of both arms of the baroreflex, vagal (BRS_+*seq*_) and sympathetic (BRS_−*seq*_), in the two groups of women. In addition, the sensitivity of both arms of the baroreflex was not affected by the menstrual phase (results of the two-way ANOVA for repeated measures revealed that both the menstrual phase–group interaction and the menstrual phase effect were not statistically significant: *F* < 1 and *P* > 0.05 for sensitivity of both arms of the baroreflex. During a 60° HUT of women with PMS, the reduction in the BRS in the LL phase was significantly more pronounced than that in the EF phase (results of the two-way ANOVA for repeated measures revealed significant menstrual phase–group and menstrual phase effect interactions: *F* = 12.1, *p* = 0.004 and *F* = 2.58, *p* > 0.05, respectively for BRS_+*seq*_, and *F* < 1.0 and *p* > 0.05 and *F* = 3.6 and *p* = 0.08, respectively for BRS_−*seq*_)

**Table 3:** Summary of the results of the power spectral analysis of heart rate (HR) and systolic blood pressure (SBP) of women without premenstrual syndrome (CON) and with premenstrual syndrome (PMS) in a recumbent position and during a 60° head-up tilt during the early follicular phase (EF) and the late luteal phase (LL) of the menstrual cycle.

	CON		PMS	
Supine	EF	LL	EF	LL
Variance, ms	5,010 ± 1,430	6,050 ± 2,500	4,720 ± 1,080	3,800 ± 1,000
LF_RR_, ms^2^	725 ± 230	665 ± 250	630 ± 170	610 ± 180
HF_RR_, ms^2^	960 ± 280	990 ± 400	786 ± 160	670 ± 150
LF_RR_/HF_RR_ ratio	1.10 ± 0.35	1.0 ± 0.23	0.82 ± 0.14	0.86 ± 0.11
LF_SBP_, mmHg^2^	0.90 ± 0.22	1.22 ± 0.20	1.77 ± 0.46	0.98 ± 0.20^∗^
BRS_−seq_, ms/mmHg	34 ± 6	28 ± 7	32 ± 4	28 ± 4
BRS_+seq_, ms/mmHg	42 ± 6	30 ± 7	27 ± 4	32 ± 7
**Head up tilt**				
Variance, ms	1,150 ± 165	1,700 ± 420	1,930 ± 640	1,380 ± 340
LF_RR_, ms^2^	244 ± 45	245 ± 55	341 ± 100	311 ± 90
HF_RR_, ms^2^	115 ± 30	125 ± 30	271 ± 82	130 ± 30^∗^
LF_RR_/HF_RR_	3.3 ± 0.9	2.5 ± 0.05	2 ± 0.45	3 ± 0.4
LF_SBP_, mmHg^2^	3.6 ± 1.2	4.5 ± 1.2	3 ± 0.5	5 ± 0.9^∗^
BRS_−seq_, ms/mmHg	7.5 ± 1	6.5 ± 0.9	10.2 ± 1.8	7.8 ± 1.3^∗^
BRS_+seq_, ms/mmHg	7.2 ± 0.7	8.4 ± 1.3	11.2 ± 1.5^#^	7.9 ± 1.1^∗^

*LF_RR_ and HF_RR_, low and high frequency domain, respectively, of the R-R intervals. LF_SBP_, low frequency domain of systolic blood pressure. BRS, baroreflex sensitivity; both the BRS_−seq_ and the BRS_+seq_ slopes represent the corresponding concordant increase (+seq) and decrease (−seq) in systolic BP and RR, respectively. ^∗^p < 0.05, paired data between the menstrual phases EF and LL into each group, ^#^p < 0.05, unpaired data between the two groups in each menstrual phase. The results of F and P values of the two-way repeated measures ANOVA are presented in the section “Results.”*

### Correlations

There were no significant correlations between the plasma E2 and P levels and the plasma NE concentrations in both groups. A significant linear correlation was found between the plasma P levels and the ISOP_15_ value in the women without PMS (*r* = 0.43, *p* = 0.035). A significant linear correlation between plasma P levels and the PHE_15_ value was found in the women with PMS (*r* = 0.44, *p* = 0.05). The plasma sex hormones levels did not correlate with any results of the power spectral analysis.

## Discussion

The main findings of this investigation are (1) plasma NE concentrations are profoundly suppressed in women with PMS during the LL phase when they are recumbent and during a 60° HUT, (2) women with PMS are less sensitive to the α_1_-adrenoreceptor agonist (PHE) in the LL phase than in the EF phase of their menstrual cycle, and the menstrual cycle has no effect on their β-adrenoreceptor responsiveness (ISOP_15_), (3) the sympathetic and vagal cardiac control in women with PMS is not affected by the menstrual phase during recumbency, and women with PMS in the EF phase have an augmented decrease in the value of HF_*RR*_ during a 60° HUT, and (4) the sympathetic vascular control (LF_*SBP*_) in the women with PMS during the LL phase is low when they are recumbent and very high during a 60° HUT, this occurs with correspondent changes in the baroreflex sensitivity.

The women with PMS in this investigation have prevalently somatic symptoms without a significant psychiatric profile and were stringently selected in order to minimize the known effects of various psychiatric disorders on the studied autonomic parameters (Landen et al., 2004; Alvares et al., 2016). This stringent selection was done because we hypothesized that the autonomic regulation of the cardiovascular system is perturbed in women with PMS (not PMDD) and this perturbed control accounts for deranged hemodynamics and their somatic symptoms.

We found that the plasma E2 and P levels were similar in the EF and LL phases of the menstrual cycle of the women with and without PMS. Although this finding is in agreement with other reports, they conflict with those of others who reported dissimilarity in plasma P levels during the luteal phase (Redei and Freeman, 1995; Rosenfeld et al., 2008). Several other studies showed that P and its metabolite, allopregnanolone (a GABA receptor modulator) during the LL phase are involved in the ethiopathogenesis of PMS.(Britton and Koob, 1998; Monteleone et al., 2000) The role of ovarian hormones and their metabolites in the pathogenesis of PMS warrants further investigation in an appropriately designed study.

### Plasma NE Concentrations

We found the plasma NE concentrations in the women with PMS were substantially lower than those in the women without PMS. It has been reported that plasma NE levels can fluctuate during a normal menstrual cycle in healthy women (Blum et al., 1992; Hirshoren et al., 2002). It also has been reported that these fluctuations are small and may not occur in women with PMS (Odink et al., 1990; Blum et al., 2004). Girdler et al. (Blum et al., 2004) reported that women with PMDD have high plasma NE concentrations. Blum et al. (Girdler et al., 1998) reported that the plasma NE concentrations in women with PMS are lower than those in women without PMS during the luteal phase of their menstrual cycle. Our findings on changes in plasma NE concentrations in two different phases of the menstrual cycle are in agreement with those of Blum et al. Previously, we reported that plasma renin activity and plasma aldosterone levels are increased during the LL phase of the menstrual cycle of women with PMS and these increases could account for their increased fluid retention (Rosenfeld et al., 2008). The increased retention of systemic fluid could contribute to the low plasma NE concentrations by a dilution effect. Of note, the plasma NE concentration is not a surrogate marker of systemic sympathetic tone, as will be detailed below (Jacob et al., 1985, 1999).

### Power Spectral Analysis

HR and BP variability are commonly used as indirect indices for evaluating autonomic control of the cardiovascular system (Pagani et al., 1986; Furlan et al., 2000). LF_*RR*_, HF_*RR*_, and especially the LF_*RR*_:HF_*RR*_ ratio are widely used indices of cardiac autonomic tone and the LF_*SBP*_ is a direct index of sympathetic vascular tone. Additionally, the latter correlates with the muscle sympathetic activity (MSNA), which is a direct marker of sympathetic vascular tone (Pagani et al., 1997; Pagani and Malliani, 2000). However, it is known that MSNA does not correlate with plasma norepinephrine concentrations (Khan et al., 2002).

We found that vagal and sympathetic cardiac control were comparable in the two groups of women when they were recumbent and during a 60° HUT. de Zambotti et al. (2013) reported that cardiac autonomic control and cardiac vagal tone are similar in the EF and LL phases of the menstrual cycle of women with and without PMS. Previously, we reported vagal predominance in cardiac autonomic control in healthy young women during the EF and LL phases of their menstrual cycles (Lavi et al., 2007). The vagal predominance in cardiac autonomic control in the LL phase of the menstrual cycle of women with PMS is controversial. Some investigators found that cardiac vagal activity declined during the luteal phase (Bai et al., 2009; Tenan et al., 2014), and others reported no changes in cardiovascular autonomic control occurred during the menstrual cycle of healthy fertile women without PMS (Leicht et al., 2003; Weissman et al., 2009; O’Donnell et al., 2015; von Holzen et al., 2016). The differences in findings can be attributed to the heterogeneity of the investigated subjects, selection bias in small cohorts and the studied menstrual phase.

It has been suggested that sympathetic cardiac control predominates in women with PMDD (Landen et al., 2004). This finding is to be expected because PMDD, as opposed to PMS, is characterized by symptoms of anxiety and depression, which affect the systemic autonomic state (Bosman et al., 2016).

During the LL phase, we found that the sympathetic tone in women with PMS, as measured by the LF_*SBP*_, was lower when they were recumbent and higher during a 60° HUT than that of women without PMS. Our knowledge on sympathetic vascular tone in healthy women with regular menstrual cycles is scant. Minson et al. (2000a) showed that the MSNA during the mid-luteal phase was higher than that in the EF phase of healthy women, while Carter et al. (Carter et al., 2009) reported that the MSNA is not affected by the menstrual phases. The results of this latter study suggest that the hormonal fluctuations which occur during a normal menstrual cycle may alter sympathetic outflow but not the transduction of sympathetic traffic into vascular resistance (Hart et al., 2011; Baker et al., 2016).

Previously, we reported that the plasma volume in the LL phase is increased in women with PMS (Rosenfeld et al., 2008). In addition to low sympathetic vascular tone in the LL phase, we can posit that the BP in the LL phase of the menstrual cycle of women with PMS depends more on their blood volume and not on their sympathetic tone when they are recumbent. Nevertheless, we found that the women with PMS need to further increase their vascular sympathetic tone in order to maintain a normal BP when they are orthostatically stressed in the LL phase.

### Baroreflex Sensitivity

BRS (gain) has a pivotal role in the regulation of BP when an individual is recumbent or erect (Eckberg and Sleght, 1992). Minson et al. (2000b) reported normal young women on oral contraception have higher sympathetic baroreflex sensitivity during low compared to the higher hormonal phase. Carter et al. (2010) and Fu et al. (2009) did not find that the BRS affected the changes in plasma sex hormone levels along the menstrual cycle. Although postmenopausal women (low E-P state) present a reduced cardiac vagal arm of BRS, healthy young women in their lowest E-P state (EF menstrual phase) show an increase in vagal arm of the BRS (Saeki et al., 1997; Lavi et al., 2007). This suggests that the BRS is affected by age rather than by sex hormone levels. We found that the vagal and sympathetic arms, the baroreflex sensitivity, were statistically not different during the menstrual phases, when recumbent. However, during orthostatic challenge, the magnitude of the reduction in the sensitivity of the sympathetic arm of the baroreflex is greater in the women with PMS than that in the women without PMS in the LL phase. This higher decrease in the sensitivity of the sympathetic arm of the baroreflex underlies the higher increase in the vascular sympathetic tone (LF_*SBP*_) during LL phase in these women.

### Responses to Adrenoreceptor Agonists

BP and HR responses to a vasoactive agent mainly depend on receptor sensitivity and baroreflex sensitivity. The diminished response to the α1-adrenoceptor agonist, PHE, in the women with PMS during the LL phase of their menstrual cycle could be due to a decrease in α_1_-adrenoreceptor sensitivity or to an increase in the sensitivity of the cardiovagal BRS (vagal arm: BRS_+*seq*_). Since we found that the cardiovagal arm of the baroreflex remains unchanged in the women with PMS during the LL phase, we can deduce that α_1_-adrenoceptor sensitivity decreases during the LL phase. The data on α1-adrenoceptor sensitivity during the menstrual cycle in healthy women are conflicting and data on α1-adrenoceptor sensitivity in women with PMS are lacking. Freedman and Girgis (2000) reported that vasoconstriction was augmented when NE, an α_1_ and α_2_-adrenoceptor agonist, was infused intra-arterially to healthy women during the LL phase. Since this augmented response was not detected when clonidine, a selective α_2_-adrenoceptor agonist, was infused, they deduced that α1-adrenoceptor hypersensitivity is present during LL phase when plasma E2 and P levels are high. Chan et al. (2001) reached a similar conclusion in their investigation on eumenorrheic women which showed that the vasoconstriction to norepinephrine was greater during late follicular phase (high plasma estrogen levels) as compared to the early follicular phase (very low plasma estrogen levels). In contrast, Bottari et al. (1983) found that α_1_-adrenoceptor sensitivity remains unchanged while α_2_-adrenoceptor sensitivity increases in the myometrium during the menstrual cycle.

We found a significant correlation between the PHE_15_ value and plasma P levels, but not with plasma E2 levels. Interestingly, this finding is in agreement with those found in an *in vitro* study on platelets: exposing platelets to estrogen reduces α-adrenoceptor density and exposing to progesterone reduces α-adrenoceptor affinity (Elliott et al., 1980; Re et al., 2002). In another study, Rheaume and Paton reported that progesterone has no effect on α-adrenoceptor sensitivity of the circular muscle of the isthmus of the rabbit oviduct (Rheaume and Paton, 1978). Additional investigations are needed to establish whether α-adrenoceptor sensitivity changes during the human menstrual cycle.

ISOP is a non-selective β-adrenoceptor agonist which activates both β_1_ and β_2_ subtypes. Stimulation of the β_2_-adrenoceptor results in vasodilation and activation of the β1-adrenoceptor has a positive inotropic and a chronotropic effect. Therefore, the chronotropic effect during ISOP administration is the result of a direct β1-mediated chronotropic effect and the sympathetic activation by β2-mediated vasodilation-baroreflex activation. Since the sympathetic BRS was comparable in both groups in the two menstrual phases, we can conclude that the response to intravenously administered ISOP is unrelated to the BRS. Since the ISOP_15_ values in the EF and LL phases of the menstrual cycle of women with PMS were similar, this result suggests that β-adrenoceptor sensitivity is unaffected by the hormonal milieu of the menstrual cycle. Moreover, this finding is different to that found in the women without PMS: the β-adrenoceptor responsiveness is reduced during the LL phase of their menstrual cycle. It has been reported that the density of β_2_-adrenoceptor on lymphocytes of women with PMDD is greater than in women without PMDD (Gurguis et al., 1998). Similar results were reported in patients with anxiety and with panic attack (Albus et al., 1986). This β-adrenoceptor hypersensitivity could explain some of the sympathetic-related symptoms during the LL phase in women with PMDD. From these findings, one can conclude that the women with PMS have “relative” β-adrenoceptor hypersensitivity, and that PMS is a mild form of PMDD.

The presence of β-adrenoceptor hypersensitivity in PMDD is attributed to both high receptor density and increased coupling of Gs-protein with cyclic AMP in the presence of high plasma P and E2 levels (Wheeldon et al., 1994). We and others have reported that β-adrenoceptor sensitivity is greater in the EF phase than in the LL phase of the menstrual cycle of eumenorrheic young women (Newnham et al., 1994; Hirshoren et al., 2002).

Collectively, our results on adrenoreceptor sensitivity in women with PMS suggest that their resting BP in the LL phase of their menstrual cycle is not dependent upon change in vascular α-adrenoreceptors sensitivity.

### Integrative Interpretation of the Results

The present study reports integrative information on the function of the autonomic nervous in women with PMS and age-matched women without PMS. Our results suggest that the symptoms and hemodynamics of women with PMS could be viewed as follows:

(1) The prevalence of autonomic-related symptoms in women with PMS is less than those reported in women with PMDD (Bosman et al., 2016). This prevalence could be explained by our finding that the perturbation in the activity of the sympathetic nervous system in women with PMS is of a small magnitude to justify stating that the system is hyperactive. Moreover, the decrease in α-adrenoceptor sensitivity and preserved β-adrenoceptor responsiveness in PMS could explain the absence of vasomotor-related symptoms, the low prevalence of palpitations and chest discomfort, much lower than reported in PMDD (Gurguis et al., 1998).
(1) Recently, it has been reported that PMS has been considered as a sentinel for future risk of hypertension (Bertone-Johnson et al., 2015). We and many others have reported that the BP during the menstrual cycle of women with and without PMS does not change despite alterations in homeostatic mechanism (Hirshoren et al., 2002; Carter et al., 2013). It emerges, and worthy to emphasize, that the homeostatic mechanisms which regulate BP in women with PMS are peculiar. Previously, we reported that women with PMS have increased fluid retention and increased activation of the renin–angiotensin–aldosterone system (RAAS) during the LL phase of their menstrual cycle (Rosenfeld et al., 2008). In this investigation, we found that the women with PMS subjects have low plasma NE concentrations, low sympathetic vascular tone (low LF_*SBP*_), and decreased α_1_-adrenoceptor sensitivity, and preserved sympathetic and vagal cardiac control and BRS during the LL phase. Therefore, we can deduce that the control of BP in women with PMS during the LL phase (compared to EF phase) is less dependent on neurovascular mechanisms, and more affected by volume regulatory systems (mainly the RAAS). However, when erect, the BP in the women with PMS becomes more dependent on the sympathetic nervous system, as demonstrated by a significant decrease in the sympathetic arm of the baroreflex, which in turn facilitates a greater increase in vascular sympathetic tone (LF_*SBP*_).

## Study Limitations

The women with PMS were selected according to stringent inclusion criteria which excluded many subjects (∼90%). These stringent criteria may restrict our conclusion to only subjects with moderate to severe form of PMS (somatic syndrome). The lack of recording MSNA may have weakened our conclusion on the central control of autonomic nervous system. Endothelial-dependent vasodilation is affected by plasma E levels and it was not considered in our study. Even though the sample size was based on power of 80% for the selected parameters, some of the results may needs a bigger sample size for confirmation. The statistical tests which were used in this investigation were selected after assessing the normality of the data (parametric vs. non-parametric) using the Kolmogorov–Smirnov goodness-of-fit test. Some investigators have criticized the use of these tests because they are based on the assumption that the samples are chosen from a normally distributed population. The symptoms were self-reported by answering a dichotomized model and not-scoring model. This self-reporting precludes testing the correlation between the results of various autonomic parameters and symptoms.

## Conclusion

The presence of autonomic symptoms in women with PMS is not associated with increased activity of sympathetic nervous system. Although the autonomic cardiovascular control is preserved during recumbency, but these women need to increase their vascular sympathetic tone to maintain normal blood pressure when they are orthostatically stressed during the luteal phase of their menstrual cycle.

## Ethics Statement

The study was approved by Institutional Ethics Committee of the RAMBAM Medical center, Haifa. The protocol was approved by the Institutional IRB. All subjects gave written informed consent in accordance with the Declaration of Helsinki (IRB-1628).

## Author Contributions

JA participated in writing the manuscript and critically revised it. RK and LD performed the experiments. GJ conceived the research, supervised experiments, and wrote the manuscript.

## Conflict of Interest Statement

The authors declare that the research was conducted in the absence of any commercial or financial relationships that could be construed as a potential conflict of interest.
